# Characterization of Antibiotic Resistance in *Shewanella* Species: An Emerging Pathogen in Clinical and Environmental Settings

**DOI:** 10.3390/microorganisms13051115

**Published:** 2025-05-13

**Authors:** Shahid Sher, Gary P. Richards, Salina Parveen, Henry N. Williams

**Affiliations:** 1 School of the Environment, Florida A&M University, Tallahassee, FL 32307, USA; shahid.sher@ufl.edu; 2 U.S. Department of Agriculture, Agricultural Research Service, Delaware State University, Dover, DE 19901, USA; gary.richards@usda.gov; 3 Department of Agriculture, Food and Resource Science, University of Maryland Eastern Shore, Princess Anne, MD 21853, USA; sparveen@umes.edu

**Keywords:** antibiotics, resistance, *Shewanella*, genes, human, efflux pump

## Abstract

Antibiotic resistance is increasing at an alarming rate worldwide, in large part due to their misuse and improper disposal. Antibiotics administered to treat human and animal diseases, including feed supplements for the treatment or prevention of disease in farm animals, have contributed greatly to the emergence of a multitude of antibiotic-resistant pathogens. *Shewanella* is one of many bacteria that have developed antibiotic resistance, and in some species, multiple-antibiotic resistance (MAR). *Shewanella* is a rod-shaped, Gram-negative, oxidase-positive, and H_2_S-producing bacterium that is naturally found in the marine environment. In humans, *Shewanella* spp. can cause skin and soft tissue infections, septicemia, cellulitis, osteomyelitis, and ear and wound infections. Some *Shewanella* have been shown to be resistant to a variety of antibiotics, including beta-lactams, aminoglycoside, quinolones, third- or fourth-generation cephalosporins, and carbapenems, due to the presence of genes such as the *bla_OXA_*-class D beta-lactamase-encoding gene, *bla_AmpC_*-class-C beta-lactamase-encoding gene, and the *qnr* gene. Bacteria can acquire and transmit these genes through different horizontal gene-transmission mechanisms such as transformation, transduction, and conjugation. The genes for antibiotic resistance are present on *Shewanella* chromosomes and plasmids. Apart from this, heavy metals such as arsenic, mercury, cadmium, and chromium can also increase antibiotic resistance in *Shewanella* due to co-selection processes such as co-resistance, cross resistance, and co-regulation mechanisms. Antibiotics and drugs enter *Shewanella* spp. through pores or gates in their cell wall and may be ejected from the bacteria by efflux pumps, which are the first line of bacterial defense against antibiotics. Multiple-drug resistant *Shewanella* can be particularly difficult to control. This review focuses on the phenotypic and genomic characteristics of *Shewanella* that are involved in the increase in antimicrobial resistance in this bacterium.

## 1. Introduction

One of the defining moments in the history of human disease treatment was the discovery of antibiotics, which have saved billions of lives. Most antibiotics are produced naturally by some species of bacteria and fungi, while others are semi-synthetic or synthetic [1,2]. The period from about 1930 to 1960 was a golden era of antibiotics due to a rise in their discovery [3]. The different classes of antibiotics are based on their modes of action, e.g., the inhibition of protein, nucleic acid, or cell wall synthesis in bacterial cells [4,5]. Apart from these, there is another mechanism in which antibiotics work to alter the biosynthetic processes in bacteria [6]. The most abundantly used antibiotic classes are penicillins, macrolides, cephalosporins, and fluoroquinolones [7].

Antimicrobial resistance is an alarming public health concern that affects the treatment of infectious diseases and the global healthcare system. Currently, resistance against antibiotics is increasing in Gram-negative bacteria at a high rate [8]. Antibiotic resistance genes (ARGs) are found in every type of environment including in the ancient sediments of permafrost [9]. The environmental reservoirs for ARGs still remain unclear [10]. *Shewanella* spp. are commonly isolated from aquatic environments that are also important reservoirs for ARGs [11,12,13]. Anthropogenic release of antibiotics and other contaminants, including heavy metals, has been reported to increase the selection of antibiotic resistance among native microbiotas, resulting in exposures of large numbers of humans and animals to antimicrobial-resistant pathogens [10,14,15]. The contamination of estuaries with these kinds of contaminants can increase the number of antibiotic-resistant bacteria as well as ARGs [16,17]. Due to increases in multiple-drug resistance (MDR) in *Shewanella* spp., it is necessary to understand the pathways and processes through which this occurs in different types of environments [18].

## 2. Background of *Shewanella*

The first report of the genus *Shewanella*, *Shewanella putrefaciens*, described its isolation from putrid butter in 1931 (Dearby and co-workers) and it was assigned to the *Achromobacter* genus [11]. Over the years, the species underwent several re-classifications and nomenclature changes. In 1941, Long and Hammer re-classified the species as *Pseudomonas putrefaciens* due to its rod shape, motility, and non-fermentative behavior [19]. This bacterium was actually *Shewanella algae*, but due to limitations at the time in biochemical identification, it was misidentified as S. *putrefaciens* [20,21]. In 1941, it was placed in *Pseudomonas* groups III and IV [22]. Due to its low G + C content (43–53%), as compared to other *Pseudomonas* species (58–72%), the *Shewanella* were assigned to the genus *Alteromonas* [11]. In 1985, MacDonnell and Colwell created a new genus for these organisms based on 5S rDNA and named it *Shewanella* in honor of Dr. James Mackay Shewan, in recognition of his contributions to the field of fishery microbiology [23,24]. At that time, *S. putrefaciens*, *Shewanella benthica*, and *Shewanella hanedai* were placed in this genus under the family *Vibrionaceae*, which has common phenotypic characteristics [25]. With the emergence of 16S rRNA sequence analysis, and better information on the relatedness among different isolates, a new family, *Alteromonadaceae*, was established, to which *Shewanella* was assigned. The family was further divided into eight families due to the large number of organisms listed, including the *Shewanellaceae* family, in which *Shewanella* is a separate genus [26]. This *Shewanella* is in the family of Gamma proteobacteria and in the order Alteromonadales. Currently, 114 species have been identified in this genus as per GenBank data accessed in November 2024.

The genus *Shewanella* has taxonomically been classified as *Pseudomonas*, *Alteromonas*, *Achromobacter*, and *Shewanella* [27,28]. The *Shewanella* spp. can be used as a potential agent of bioremediation against halogenated organic compounds [29]. *Shewanella algae*, being an oppurtunistic pathogen, frequently found in aquatic ecosystems, has been shown in various clinical cases in people who ingest raw seafood and are exposed to the marine environment [30]. Morover, it has been implicated in infective arthritis, infective endocarditis, peritonitis, and ear and eye infection [30]. *S. xiamenensis* is a zoonotic pathogen and can cause intra-abdominal infections in human beings [31], *S. haliotis* can cause severe soft tisse infection [32] and S. upenei can cause bacteremia and lung infections [33]. *Shewanella putrefaciens*, isolated from food and environmental samples, is a human pathogen that has been isolated from cases of septicemia (a combination of bacteremia and toxemia) [18]. It is a rod-shaped, Gram-negative, oxidase-positive, and H_2_S-producing bacterium [34]. It was first isolated in 1995, from rabbit fish, *Siganus rivulatus*, and has been implicated as a cause of fish spoilage [35]. The role of genes in antibiotic-resistant pathogens in aquatic environments is a matter of great concern. Antibiotics (tetracyclines, sulfonamides, and fluoroquinolones) can persist in soil and aquatic environment for several months [36]. As a result, their residues can affect the communities of microorganisms in sediments and water bodies and give rise to some microorganisms that contain ARGs for the respective antibiotics [37,38]. The existence of antibiotic resistance in *S. putrefaciens* has been reported in marine environments and can be lethal to marine life such as shellfish and other marine organisms [39].

## 3. Microbiological Characteristics of *Shewanella*

*Shewanella* is a Gram-negative bacterium with a pleomorphic morphology, and it has a length of 1–3 µm and a diameter of 0.5–0.65 µm. It has a straight or curved rod shape with a polar flagellum [40] and does not form spores [22]. The bacteria of this genus are chemoorganotrophic, catalase positive, oxidase positive, can degrade gelatin, and reduce nitrate into nitrite. *Shewanella* are aerobic or facultative aerobic bacteria. Some species can ferment carbohydrates such as D-glucose and, typically, produce acid without gas formation. Some, but not all, species are H_2_S positive. API 20E can be used for the identification of *Shewanella* spp., but in one study 46.8% of bacterial isolates from water and shellfish samples could not be identified on the API 20E database identification system [41]. Some species are psychrophiles and can grow at 4 °C. On growth agar media such as Luria–Bertani agar (Sigma-Aldrich, L3147, St. Louis, MO, USA), Tryptic soy agar (HiMedia Laboratories, M290, Mumbai, MH, India), marine agar (Difco/Becton Dickinson, 2216, Franklin Lakes, NJ, USA), and iron agar (HiMedia Laboratories, M021, Mumbai, MH, India), *Shewanella* colonies can change color from pale tan to salmon color or orange pink due to the high accumulation of proteins such as cytochrome. On iron agar, it produces black colonies due to iron reduction (Fe^3+^ to Fe^2^). The color change may not be observed when cytochrome production is low. The culture age and type of medium influences the color change in species, such as in *S. colwelliana* and *S. hanedai*, which can show dark-brown pigments on media amended with L-tyrosine. *Shewanella algae* and *S. colwelliana* grow well in media containing seawater due to their requirement for sodium ions [42].

## 4. Molecular Identification/Characterization of *Shewanella*

Molecular methods such as sequence analysis of 16S rRNA and *gryB* gene (encoding the B subunit of the DNA gyrase enzyme) and DNA hybridization have been used to improve species-level identification of *Shewanella* isolates; 16S rRNA is used most to identify *Shewanella* spp. For the discrimination of closely related species, *gyr*B gene sequence analysis works well [43,44]. For the identification of new *Shewanella* spp., DNA-DNA hybridization has been reported to work well [22,45]. The GC content in *Shewanella* spp. can be measured by analyzing WGS, values for which are available on the NCBI database. Estimates can also be determined by the thermal renaturation method (Tm) and HPLC. The GC content ranges between 38% and 54%. To date, there are 101 reference genome sequences available in the NCBI database for *Shewanella*, representing different species having different numbers of genome assemblies. The maximum number of genome assemblies available for the genus *Shewanella* is 915, which has not yet been characterized for the species level. The first reported genome sequence for *Shewanella* was *S. oneidensis*, sequenced in 2002. This species, which is a potential bioremediation organism, has one plasmid along with a single chromosome. Currently, there are five genome assemblies for this bacterium deposited at the NCBI. The two *Shewanella* species that are most clinically important are *S. putrefaciens* and *S. algae*, with genome assemblies of 22 and 230, respectively. The genome assemblies and their sizes for different *Shewanella* species are shown in Table 1.

## 5. Heavy Metals’ Roles in Antibiotic Resistance Acceleration

Many studies have documented that heavy metal exposure can increase the transmission of antibiotic resistance through the process of co-selection [46,47]. There are different mechanistic roles in co-selection such as co-resistance, cross resistance, and co-regulation [47]. The process of co-resistance occurs when genes for both heavy metal and antibiotic resistance are in proximity to each other in the same promotor region on the bacterial genome [48]. It has been reported that plasmids for co-resistance may be present in bacteria isolated from humans and in some domestic animals [49]. Another mechanism is cross resistance, which occurs when a single system (e.g., efflux pump) confers resistance to both heavy metals and antibiotics. This efflux system is most common in *Shewanella* spp. [50]. The efflux pump for multiple drugs is common and contains well-conserved elements in microorganisms, which efflux or pump antimicrobial substances from the cytoplasm of the bacterial cell to the outer environment [51]. These compounds include antibiotics, metals, plant-produced compounds, quorum sensing molecules, and bacterial metabolites and by-products [52]. It is challenging for scientists to determine the regulatory mechanism for these MDR efflux pumps and how they can be regulated. The role of the efflux pump in promoting MDR in *Enterobacteriaceae* has been reported [53]. The third mechanism through which co-selection can occur is co-regulation, in which the resistance genes for metals and antibiotics are co-regulated by the process of transcription. In *Shewanella* and *Vibrio* strains, genes for co-selection phenotypes are on the chromosomes. The homologous gene for the efflux pump indicates that cross resistance and co-regulation contribute to resistance against metals/antibiotics [54].

Bacteria that are resistant to arsenic and mercury are more resistant to antibiotics than bacteria that are sensitive to these metals [55]. In a study of the intestinal microbiota of mummichog (a small killifish found along the Atlantic coast of Canada and the US), *Shewanella* spp. made up a sizable fraction of the intestinal flora. Analysis of the whole genome sequence of *Shewanella* found that the genes for heavy metals and antibiotic resistance are co-selected by the same promoter region [54]. *Shewanella* spp., such as *Shewanella putrefaciens* and *Shewanella algae*, are opportunistic pathogens that can cause human infections and serve as a vehicle for MDR, which is a public health concern because of ARGs [11,56].

## 6. Antibiotic Reservoirs in the Aquatic Environment

Antibiotic resistance is increasing on a global scale and at an alarming rate for bacteria found in humans and other organisms [57]. The aquaculture sector cannot be ignored when considering the reservoirs of antibiotic resistance [58]. Veterinary medicine is the major cause for the increase in antibiotic usage in aquaculture, where antibiotics are used for the treatment of illnesses in animals, disease prevention, and promoting the growth of animals. *Shewanella* spp., including some antibiotic-resistant strains, are naturally found in the marine environment and may be a reservoir for antibiotic-resistant genes. They can be isolated from seawater, marine sediments, fish, shellfish, animals, sand, and soil [41,59]. Some *Shewanella* spp. have been reported to show resistance to β-lactam antibiotics and carbapenems, which disrupt the integrity of the bacterial cell wall, and are used to treat hospital-acquired infections of Gram-negative pathogens [60]. Human infections by *Shewanella* spp. resulting from direct and indirect contact with seawater or seafood are sporadic and opportunistic [61,62].

The whole genome sequence of a multi-drug-resistant *Shewanella* sp. isolated from the gastrointestinal tract of mummichog fish (*Fundulus heteroclitus*) was analyzed [54]. Phenotypically, the isolated strain showed resistance to various *β*-lactam antibiotics and to multiple metals including mercury and arsenic, but the genome analysis only detected the resistance gene for carbapenemase (blaOXA-48). This indicates that the phenotypic co-selection of antibiotics and metals is regulated by genes present on chromosomal DNA. Additionally, the homologs of efflux pump genes were identified and may play a role in the efflux of antibiotics to convey bacterial resistance to the bacterium. Based on that study, it was suggested that the gut microbiota of mummichog fish could be a reservoir for clinical strains of antibiotic resistant *Shewanella* spp.

## 7. *Shewanella* in Human Infections

*Shewanella* is present in sediment and aquatic environments [63]. Although human infections due to *Shewanella* spp. are rare, the bacteria of this genus are potential emerging pathogens, and in recent decades have received attention because of the emergence of antibiotic resistance in some species, complicating treatment with traditional antibiotics [18]. Most of the bacterial strains belonging to *Shewanella* spp. produce hemolysis of red blood cells, which are potential signs of *Shewanella* pathogenicity [13,64,65,66]. *Shewanella* species have been reported to cause skin and soft tissue infections, and septicemia [67,68]. Other diseases in which *Shewanella* have been reported include hepatobiliary disease, otitis media, and pneumonia [69]. In immunocompromised individuals with low immunity, renal failure, neutropenia, hepatobiliary disease, and diabetes can be associated with *Shewanella* infection [18,40,69].

Several clinical studies of *Shewanella* infections in hospitals have been reported. In one such study conducted in a regional hospital in Hong Kong, 128 patients were identified with *Shewanella* species infections over a 10-year period between April 2010 and December 2020 [40]. Among the patients, 61.7% were male in the age range between 65 and 87. *Shewanella algae* accounted for 92.2% of the infected patients, with 7.8% infected with *Shewanella putrefactions* [40]. More than 93% of isolates were sensitive to ceftazidime, gentamicin, and ciprofloxacin and 76.6% isolates were sensitive to imipenem. In a study at the University Hospital of Gran Canaria, Spain, 31 patients with *Shewanella* infections were recorded between 2001 and 2016 [43]. Of these, most were males, with eight females. The patients’ age ranged between 15 and 87, with a mean of 50.7 years. Fifteen of the patients were infected with *S. putrefactions* and sixteen were with S. algae. Eighteen isolates were obtained from skin and soft tissue infections, with eight from blood infections, two from peritoneal lesions, one from bronchial aspirate, one from bile, and one from an ear swab [42]. In a study of a retrospective analysis of medical and laboratory examinations of patients infected with *Shewanella* species over the last 10 years published in 2024 [70], 68.8% of 51 cases were identified as *S. putrefactions* and 31.4% as *S. algae*. The isolates were resistant to ticarcillin-clavulanic acid (23.5%), cefoperazone-sulbactam (19.6%), cefotaxime (17.6%), and ciprofloxacin (17.6%).

Infections due to *Shewanella* can be treated with a variety of antibiotics, including β-lactams, aminoglycosides, and quinolones, third- or fourth-generation cephalosporins, carbapenems, β-lactamase inhibitors, aminoglycosides, chloramphenicol, erythromycin, and aztreonam [11,19,22]. Bacteria have acquired some resistance genes that have made some of these antibiotics less effective [71]. These include the blaOXA-Class D β-lactamases encoding gene, *bla_AmpC_*-class-C β-lactamases encoding gene, and *qnr* gene, which confer resistance against different antibiotics such as carbapenems, cephalosporin, and quinolones [43]. The last-resort antibiotic in human medicine, colistin or polymyxin, used to treat MDR bacteria, is becoming ineffective against *Shewanella*-related infections due to the presence of the chromosomal *eptA* gene, which encodes for lipid-A phosphoethanolamine transferase [44]. This enzyme changes the lipid A portion of lipopolysaccharide/lipooligosaccharide by the addition of phosphoethanolamine, which results in the reduction in the overall negative charge of the outer membrane. The reduction in overall negative charge reduces the ability of cationic antibiotics to bind effectively with the outer membrane of the bacteria [58].

In recent years, there has been more attention focused on antibiotic resistance genes in the genus *Shewanella* due to its potential role in the transmission of antibiotic resistance determinants or genes [58]. Genes for antibiotic resistance have been detected in *S. algae*, *S. putrefaciens*, *S. xiamenensis*, *S. oneidensis*, and *S. frigidimarina* [11]. Initially, these resistance genes were identified on chromosomes in *Shewanella* spp., but not on mobile genetic elements (MGEs). However, recent studies have detected multiple resistance genes in MDR plasmids that are different from integrons and transposons, the mobile genetic elements [58]. A recent review of *Shewanella* covered the microbiological and infectious aspects of these bacteria [34]. The present review here will provide information regarding various aspects of the genus *Shewanella*, but with a strong focus on antimicrobial resistance elements and mechanisms.

## 8. Mechanisms of Antibiotic Resistance

**Figure 1:**
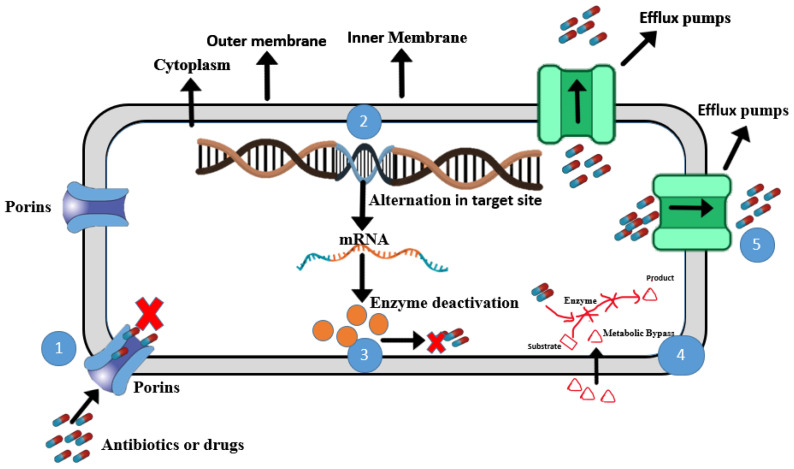
Six antibiotic resistance mechanisms in *Shewanella*. (1) **Porins:** alternation in porins limits entrance of antibiotics in cell. (2) **Alteration in targeted sites:** antibiotic no longer binds with altered targeted site. (3) **Enzyme deactivation:** intracellular enzymes in bacteria modify antibiotics. (4) **Metabolic bypass:** bacteria produce enzymes which bypass antibiotic action. (5) **Efflux pump:** actively transports antibiotics from inside to outside of cell to prevent antibiotic accumulation in the cell. (Figure created by ChemBioDraw, ultra version 13).

The different bacterial resistance mechanisms against antibiotics are shown in Figure 1.

**Porins:** The cell membrane of Gram-negative bacteria plays a role in their survival and environmental adaptation. Porins are small holes or channels in the outer membrane of Gram-negative bacteria that regulate the influx of nutrients and antibiotics into the cell [72]. Some porins are very specific, such as LamB and FepA, which are involved in the uptake of maltose and iron, respectively [72]. Antibiotics gain access to targets inside the cell through porins. If the porins are modified (changed), closed, or reduced, antibiotics cannot enter the cell [73]. There are five common porin proteins—OmpA, OmpC, OmpF, OmpW, and OmpX—that have role in porin permeability [73]. In *Shewanella oneidensis*, the role of major porins remains unexplored. Two porins, OmpS38 and OmpA, do not influence the natural resistance to β-lactam antibiotics [74]. A PacBio sequence analysis in *S. algae* revealed that *pdsS* is a sensor histidine kinase which is further associated with pdsO (outer membrane-like protein). These *pdsO* proteins have a role in membrane integrity and beta-lactam antibiotic resistance [75].

**Alterations in targeted sites:** Antibiotics have very specific target sites in bacterial cells. Alteration or mutation in these target sites can reduce the antibiotic’s ability to attach. In *Enterobacteriaceae*, the resistance against quinolones is due to a mutation in the genes *gyrA* and *parC*, which encode DNA gyrase and topoisomerase II protein [76]. These DNA gyrase and topoisomerase II are the target sites for fluoroquinolones antibiotics. Mutation in the genes *gyrA* and *parC* can alter the respective proteins. As a result, fluoroquinolones cannot properly bind with the protein and the antibiotic becomes ineffective [76]. The alteration in target sites often occurs due to spontaneous mutation in bacterial genes present on the chromosome as, e.g., in the mutation in RNA polymerase and DNA gyrase resulting in the resistance to the rifamycins and quinolones, respectively [77]. In one of the studies regarding *S. algae* strains, a mutation in the chromosome-encoded gyrase subunit (GyrA) led to resistance against quinolone antibiotics such as ciprofloxacin [78]. Qnr, a quinolone resistance protein, binds with topoisomerases (a target site for quinolones), protecting them from quinolone antibiotics [79]. This resistance mechanism is not well studied in *Shewanella* spp. and could be a valuable area for further research by microbiologists.

**Enzyme deactivation:** Extra-cellular and intra-cellular enzymes can also play a part in antibiotic resistance mechanisms in *Shewanella*. β-lactamase production in Gram-negative bacteria deactivates β-lactams and *Shewanella* species serve as a reservoir of antibiotic resistance by producing β-lactamases against β-lactam antibiotics [80]. In *Shewanella xiamenensis* strains, the (AAC(6′)-Ib-cr) protein can deactivate the quinolones by acetylating the amino nitrogen on their piperazinyl ring [79]. The *qnrA* gene in *S. algae* is located on chromosomal DNA, and this species acts as a reservoir for the *qnrA* gene, leading to a four-to-eight-fold increase in resistance against fluoroquinolone antibiotics, as measured by MIC [76]. Another study found that resistance genes for quinolone (*qepA* and *oqxAB*) are present on plasmids and these encode intracellular enzymes which expel the quinolones [79]. The probable progenitors of *bla*_OXA_-_48_-like genes are present on chromosomes and encode for beta-lactamases in *Shewanella* species, but their dissemination is unclear [80]. *Shewanella oneidensis* contains seven genes expected to encode β-lactamases, including AmpC and class D β-lactamase BlaA (also referred to as OXA-54). BlaA, along with its analog from *S. xiamenensis* (OXA-181), has been proposed as the progenitor of carbapenem-hydrolyzing oxacillinases, which enhance the effectiveness of carbapenems in clinically related Gram-negative pathogens [81,82]. While the physiological relevance of AmpC is yet to be determined, BlaA is the key enzyme conferring *S. oneidensis* resistance to some β-lactams [83]. The *S. oneidensis blaA* gene is inducible by ampicillin at high levels. Like the *ampC* genes in other enterobacteria, the *bla*A is different from *amp*C in gene regulation mechanisms. Also, the gene encoding an AmpR homolog is absent in the *S. oneidensis* genome, linked to the probability of an uncommon regulatory mechanism [83]. Siderophores, iron-binding molecules secreted by bacteria, have been shown to be involved in the transport of sequestered toxic metals and the modulation of antibiotic activity [84]. *Shewanella oneidensis* MR-1 can degrade chloramphenicol along with the metal oxide due to the presence of antibiotic resistance genes that encode specific degrading enzymes [85]. Another study found that *Shewanella* spp. can express multiple resistance genes, including beta-lactamases and aminoglycoside modification enzymes, and can sequester iron from the environment, which could be an important virulence factor in *Shewanella* spp. [77].

**Metabolic bypass:** Bacteria develop resistance to antibiotics like sulfonamides and trimethoprim by acquiring genes encoding drug-insensitive variants of dihydrofolate reductase (DHFR) and dihydropteroate synthase (DHPS), fundamental enzymes in the folate biosynthetic pathway. Such mechanisms allow bacteria to bypass the inhibitory effects of these antibiotics [86]. Metabolic bypass can play a role in the antibiotic resistance of *Shewanella* species. A study proposed that under anaerobic or oxygen-limited conditions, *S. oneidensis* MR-1 utilizes the serine-isocitrate lyase pathway, common to many methylotrophic anaerobes. In this pathway, formaldehyde is produced from pyruvate and condenses with glycine to form serine, which then enters the TCA cycle. This adaptation indicates metabolic flexibility that could potentially bypass certain antibiotic targets [87]. A multi-omics approach revealed that *S. algae* downregulate genes involved in amino acid metabolism and ubiquinol-8 biosynthesis while upregulating virulence genes. Additionally, the “one-carbon pool by folate” pathway was significantly enriched in *E. coli* OP50 compared to *S. algae*, suggesting differences in folate metabolism that could influence antibiotic susceptibility [88].

**Figure 2:**
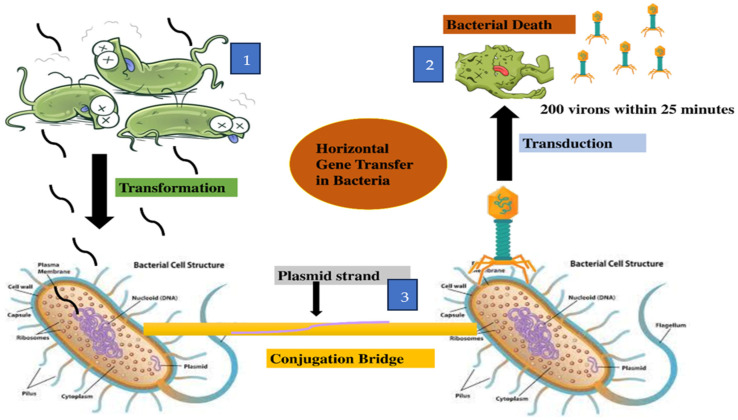
Three mechanisms for the transmission of antibiotic resistance genes in *Shewanella:* (1) **transformation:** bacterial cells taking DNA from surroundings; (2) **transduction:** bacteriophages being released after the death of a phage-infected bacterial cell; (3) **conjugation:** genetic material being transferred from one bacterium to another via a conjugation tube. (Figure created by ChemBioDraw).

**Efflux Pump:** The efflux pump is a resistance mechanism by which bacteria pump out antibiotics or drugs that have already entered inside the cell. These may be the first line of defense against antibiotics in some bacteria. The presence of efflux pumps in MDR *S. algae* can be identified by WGS analysis [59]. There are five different structural classifications or families of efflux pumps enabling MDR in these bacteria, which include the adenosine triphosphate (ATP)-binding cassette (ABC) superfamily, MTE family (multidrug and toxic compound extrusion), SMR (small multidrug resistance) family, MFS (major facilitator superfamily), and the RND (resistance/nodulation/division) superfamily [11,63,65]. All these families work to export drugs or antibiotics from inside the cell to the outside. The efflux pump is common in Gram-negative bacteria, including *Shewanellaceae*. In *Shewanellaceae*, integrative and conjugative elements (ICEs) belong to the SXT/R391 family, and mobile genes associated with antimicrobial resistance, heavy metal resistance, and virulence are the main mobile elements that are contributing to the transmission of antibiotic resistance to other bacteria in the environment. Cimmino et al. studied the resistance genes and virulence factors of *S. algae* strain MARS 14 through whole genome sequencing. This study revealed that the bacteria contained several efflux pumps for multiple-drug resistance and contained beta-lactamases class C and D, along with genes for hemolysins and biofilm formation [59]. In *S. oneidensis* strain MR-1, the *mexF* gene encodes the MDR pump, which provides resistance against tetracycline and chloramphenicol [78]. The expression of *mexEF* operon is regulated by the TetR regulatory protein and the presence of *mexF* indicates that the bacteria expel antibiotics, which is necessary for their survival [78]. As with many other bacteria, *Shewanella* antibiotic resistance genes can be transferred by three different mechanisms: transformation, transduction, and conjugation [63]. These mechanisms can occur in aquatic environmental bacteria and in human pathogenic bacteria, as shown in Figure 2.

## 9. Transmission of Resistance Genes

There are three different mechanisms through which antibiotic resistance genes get transferred in the *Shewanella* genus. These mechanisms include transformation, transduction, and conjugation. Most recently, a very significant alternation was observed in the resistance pattern of *S. xiamenensis*. These resistance genes were evaluated through illumina sequencing, which showed that bacteria acquired these genes externally. Twenty-one strains of *S*. *xiamenensis* were declared as MDR, with 10 different kinds of antibiotic resistance genes with mobile genetic elements that can disseminate resistance genes into the environment [89]. In 15 different genomes of *S. xiamenensis* strains, a type IV secretion system was discovered, each with several sequence structures, suggesting it originated from different donors through horizontal gene transfer, which include transformation, transduction, and conjugation [89]. In transformation, bacterial cell lysis results in DNA being released into the environment, including aquatic bodies. The DNA may contain ARGs. This exogenous DNA may be taken up by other bacteria present in the surrounding environment where the recipient bacteria, if naturally competent, can integrate the exogenous DNA into their chromosomes. *Shewanella baltica* electrocompetent cells were prepared and transformation of P1 and ColE1-like plasmids in *S. baltica* showed that the plasmid’s stability was lower without antibiotic selection pressure [90]. The transformation efficacy in *S. baltica* can be estimated by taking the number of *S. baltica* colonies on antibiotic agar plates, with and without antibiotic stress. It was observed that the transformation efficiency was two to three times lower than in *E. coli* [90]. Groh et al. showed that mini-Tn*10* transposon-bearing plasmid, pBSL180, was transformed, and observed that it can efficiently and randomly mutagenize *S. oneidensis* strain MR-1 [78]. The probable progenitors of *bla*_OXA_-_48_-like genes are present on chromosomes and encoded beta-lactamases in *Shewanella* species, but their dissemination is unclear [80].

The second mechanism for gene transmission is transduction, in which bacteriophages or bacterial viruses play a significant role in shaping and regulating communities of bacteria [91]. In this process, bacteriophages may contain ARGs and other genes that play a role in virulence and pathogenicity. In the infection process, the phages may take fragments of such genes from the host bacterium and transfer them to the next bacterium it infects. As a result, the recipient bacterium becomes resistant to the antibiotics. In China, from Qingdao sewage, *Shewanella KR11*-infecting phage vB_SbaS_Y11 was isolated. This phage has an icosahedral-shaped head with a long tail, as revealed by transmission electron microscopy. This phage may play a role in transduction of genes in *Shewanella KR11* [92].

The third process of gene transmission is conjugation, in which Gram-negative bacteria attach by their cell surface pili to recipient cells. The pili forms a bridge or conjugation tube through which the donor cell transfers to a plasmid, which contains integrons, insertion sequences, or transposons carrying ARGs, that confer resistance to the recipient bacterial cell. In 91 strains of *Shewanella* collected from various samples, SXT/R391 integrative conjugative elements (ICEs) were detected in different species [93]. In the same study, different antibiotic genes such as *strB*, *floR*, *strA*, and *sul2* were detected in ICE*Sup*CHN110003 (101). Conjugation does occur in aquatic environments [94].

A study conducted in Algeria identified plasmid pSx1 as having a size of 268.4 kb in the widely drug-resistant *Shewanella xiamenensis* T1 isolated from hospital waste. The isolated bacterium contained ARGs and the novel Tn*1696* derivative (Tn*6297*), a transposon that carries a resistance gene for carbapenems, and *bla*_OXA-48_-like gene (*bla*_OXA-416_) on the plasmid and chromosome [11]. Initially, the ARGs were identified by WGS analysis of *S. xiamenensis* T1. Subsequently, the resistance genes were electroporated into an *E. coli* competent strain for confirmation [11]. In *Shewanella xiamenensis*, the *qnrA1* gene was found through DNA hybridization and it is encoded on chromosomes. It is part of a class 1 integron, followed by IS*CR1*. This integron is closely related to In825, which has four genes’ cassettes, *aacA3*, *catB11c*, *dfrA1z*, and *A2az*. IS*26* was also found in a genome which provides resistance against macrolides. The presence of resistance genes in *S. xiamenensis* suggests that this species could serve as a vehicle for the dissemination of resistance genes in other bacteria [95].

*Shewanella xiamenensis* NUITM-VS2, showing resistance to tigecycline and carbapenem, was isolated from a drainage system in Vietnam in 2012 [61]. It also showed resistance to multiple antibiotics such as aminoglycosides, cephalosporins, carbapenems, and fluoroquinolone. Genome sequencing revealed one chromosome and five plasmids. ResFinder (database for ARGs) and Plasmid Finder were used for the identification of ARGs and plasmid replicons inside the genome of the isolate. *tet(X4)* and *tmexC3*.2D3.2-toprJ1, the tigecycline resistance genes, are present on the IncC plasmid pNUITM-VS2_2 (152.2 kb). Similarly, a 24.8 kb untypeable plasmid, pNUITM-VS2_4, contained the carbapenemase blaND-1 gene [61]. By conjugation, pNUITM-VS2_2 was transferred to *E. coli* and conferred high-level resistance to numerous antibiotics, including tigecycline. This discovery of RND-type efflux pump gene cluster *tmexCD*-*toprJ* in *Shewanella* species was first reported by Dao and co-workers [57].

Another study reported that different strains of *Shewanella algae* (353M, 178CP, 146bCP, 144bCP, 219VB, 82CP, 38LV, 57CP, 60CP, and 83CP) were resistant to multiple antibiotics, including amoxicillin (AMX); amikacin (AKN); cephalothin (CEF); colistin (CS); Fosfomycin (FOS); cefoxitin (CX); imipenem (IPM); and sulfonamides (SUL) [63].

An analysis of a colistin-resistant clinical isolate of *S. algae* strain MARS 14, obtained from the broncho-alveolar lavage of a hospitalized patient, was performed to determine the molecular basis for the resistance. A functional genomics method was employed in which *E. coli* TOP10 was used to construct a genomic expression library based on the pZE21 MCS-1 plasmid. The library’s estimated size was 1.30 × 10^8^ bp. LB-agar with 8 mg/L of colistin was used for the functional screening of colistin-resistant clones. Then, the genomic expression library was thoroughly screened and five clones that were resistant to colistin were obtained. Following examination of DNA sequencing results of these colonies using the Integrated Microbial Genomes (IMG) and KEGG databases, an analysis of this unique gene’s amino acid sequence revealed that it encodes for ethanolamine phosphotransferase (*EptA*, or *PmrC*). Overexpression of *EptA*, an essential enzyme for the arrangement of outer membrane lipopolysaccharides, was observed to be related to resistance to colistin in *S. algae* MARS 14 (27-fold increase) [69].

Apart from antibiotic resistance, some *Shewanella* spp. also show resistance to heavy metals such as Cd, Cr, Cu, Co, and Zn, which are toxic for many organisms. It has been reported that two (SY1 and SY2) of fifteen strains were resistant to metals and to antibiotics, including gentamicin, rifampicin, erythromycin, vancomycin, cephalothin, ampicillin, and streptomycin [96]. The genes for antibiotics and heavy metals are usually present on bacterial plasmids. There is good evidence that the development of antibiotic resistance may be associated with selective pressure from heavy metal contamination, suggesting a process of co-resistance [97,98]. Bacteria play a role in bioaccumulation, bioabsorption, and biotransformation of heavy metals in aquatic and other environments [99]. The structure and function of microbial communities can be modified or altered in the presence of these heavy metals. Bacteria may play a role in metal bioaccumulation, bioadsorption, and biotransformation of heavy metals in aquatic and other environments.

## 10. Conclusions

*Shewanella* spp. are ubiquitously distributed across environments, including aquatic ecosystems. Some species can cause infections in humans, which may be difficult to treat due to antibiotic resistance. ARGs are part of mobile genetic elements (MGEs) such as integrons, and insertion sequences present on chromosomal DNA and plasmids. *Shewanella* can acquire or transmit these genes through the process of transformation, conjugation, and transduction. The presence of these ARGs and efflux pumps supports the hypothesis that *Shewanella* is an emerging pathogen for humans with the ability to develop resistance against various types of antibiotics. There is a need for more research to evaluate other virulence factors and genes that may play an important role in diseases in humans and in aquatic animals such as oysters and shellfish. This can be better evaluated by using whole genome sequencing supported by transcriptomics and proteomics analyses to detect the products of ARGs.

## Figures and Tables

**Table 1:** Genome assemblies and their sizes in different *Shewanella* spp., all with 1 chromosome (NCBI search from 2024), https://www.ncbi.nlm.nih.gov/datasets/genome/ (accessed on 10 January 2024). ND means not determined.

Shewanella Species	Genome Assemblies	No. of Chromo-Somes	No. of Plasmids	Genome Size (Mb)	GC (%)	No. of Genes (Avg)	No. of Proteins (Avg)	First Genome Release Date
***Shewanella putrefaciens***	22	1	3	4.38–5.05	44.30–47.90	4055	3820	01/10/2014
***Shewanella algae***	230	1	1	4.60–5.20	52.50–53.2	4185	4450	04/01/2014
***Shewanella baltica***	58	1	4	3.26–5.55	45.99–47.50	4414	4587	02/20/2007
***Shewanella benthica***	4	1	ND	4.03–5.71	45.8–46	3431	3259	11/28/2007
***Shewanella woodyi***	4	1	ND	5.82–5.94	43.60–43.70	4874	5085	03/13/2008
***Shewanella halifaxensis***	2	1	ND	5.23–5.46	42.60–42.80	4674	4494	02/07/2008
***Shewanella oneidensis***	7	1	1	3.71–5.13	45.93–46.50	4438	4261	09/12/2002
***Shewanella colwelliana***	8	ND	ND	4.47–4.81	45.30–45.60	4135	4014	01/10/2014
***Shewanella vesiculosa***	12	1	ND	4.46–4.78	41–60–41.70	4068	3840	11/19/2018
***Shewanella chilikensis***	16	1	ND	4.39–4.91	52.20–52.50	3945	3845	12/12/2017
***Shewanella indica***	15	1	ND	4.38–4.99	52.20–52.60	4089	3897	09/11/2020
***Shewanella profunda***	1	1	ND	4.74	44.9	4080	4303	05/09/2022
***Shewanella glacialimarina***	1	1	ND	4.46	41.1	3763	3915	10/15/2021
***Shewanella algicola***	3	ND	ND	4.85–4.97	42.30	4267	4459	09/11/2020
***Shewanella saliphila***	2	ND	ND	4.62–4.63	42.50	3926	4086	09/11/2020

## Data Availability

No new data were created or analyzed in this study. Data sharing is not applicable to this article.
